# Race and Genetics in Congenital Heart Disease: Application of iPSCs, Omics, and Machine Learning Technologies

**DOI:** 10.3389/fcvm.2021.635280

**Published:** 2021-02-17

**Authors:** McKay Mullen, Angela Zhang, George K. Lui, Anitra W. Romfh, June-Wha Rhee, Joseph C. Wu

**Affiliations:** ^1^Stanford Cardiovascular Institute, Stanford University, Stanford, CA, United States; ^2^Department of Physiology, Morehouse School of Medicine, Atlanta, GA, United States; ^3^Department of Genetics, Stanford School of Medicine, Stanford University, Stanford, CA, United States; ^4^Department of Medicine, Division of Cardiovascular Medicine, Stanford University, Stanford, CA, United States; ^5^Department of Pediatrics, Division of Pediatric Cardiology, Stanford University, Stanford, CA, United States; ^6^Department of Radiology, Stanford University, Stanford, CA, United States

**Keywords:** congenital heart disease, iPSC, disease modeling, genomics, race, disparity

## Abstract

Congenital heart disease (CHD) is a multifaceted cardiovascular anomaly that occurs when there are structural abnormalities in the heart before birth. Although various risk factors are known to influence the development of this disease, a full comprehension of the etiology and treatment for different patient populations remains elusive. For instance, racial minorities are disproportionally affected by this disease and typically have worse prognosis, possibly due to environmental and genetic disparities. Although research into CHD has highlighted a wide range of causal factors, the reasons for these differences seen in different patient populations are not fully known. Cardiovascular disease modeling using induced pluripotent stem cells (iPSCs) is a novel approach for investigating possible genetic variants in CHD that may be race specific, making it a valuable tool to help solve the mystery of higher incidence and mortality rates among minorities. Herein, we first review the prevalence, risk factors, and genetics of CHD and then discuss the use of iPSCs, omics, and machine learning technologies to investigate the etiology of CHD and its connection to racial disparities. We also explore the translational potential of iPSC-based disease modeling combined with genome editing and high throughput drug screening platforms.

## Introduction

CHD is the most frequently occurring birth defect, and despite technological advances in healthcare, children afflicted with CHD continue to face significant morbidity and mortality (1, 2). Moreover, it remains largely unknown why certain populations (e.g., racial minorities) are disproportionally affected by this disease. Herein, we first review the prevalence, risk factors, and genetics of CHD. We then discuss the applications of iPSCs, omics, and machine learning technologies to better understand disease mechanisms especially in connection to racial disparities.

## Congenital Heart Disease: Definition, Classifications and Prevalence

Cardiac malformations present at birth make up a relevant component of pediatric cardiovascular disease that constitutes a significant percentage of clinically significant birth defects, occurring in about 4 to 50 per 1,000 live births (3). CHD is defined as a gross structural abnormality of the heart or intrathoracic great vessels arising before birth with potential functional significance (4). It is the most frequently diagnosed congenital defect among newborns and has consistently been a primary cause of morbidity and mortality among those affected (5, 6). It is estimated that 4 to 10 live-born infants per 1,000 are diagnosed with CHD in the first year of life (3, 7, 8). Additionally, 1 in 4 infants with critical CHD require surgery in the first year of life (9).

CHD can be classified either as simple, moderate, or complex based on survival, prognosis, and frequency of complications. Simple CHD typically does not require extensive surgery to repair. Common instances of simple CHD defects include isolated congenital valve disease (e.g., bicuspid aortic valve), mild pulmonary stenosis, and minor atrial septal defect (ASD) or ventricular septal defect (VSD) (10, 11). Moderate CHD requires expert care to repair to achieve better prognosis/survival compared to complex CHD (4). Examples of moderate CHD include coarctation of the aorta, Ebstein anomaly, and more complex ASD/VSD (4). Complex CHD typically presents early with profound hypoxemia and/or hemodynamic complications that generally require early interventions. Examples of frequently occurring complex CHD include complex tetralogy of Fallot (ToF), transposition of great arteries, and hypoplastic left heart syndrome (HLHS). ToF is a common cardiac defect that accounts for 5.4% of all CHDs and about 60% of conotruncal defects, excluding transposition of great arteries (12–14). It is characterized by right ventricular outflow tract obstruction, VSD, overriding aorta, and right ventricular hypertrophy. HLHS patients present with abnormally severe underdevelopment of the left ventricle, mitral valve, and aorta (15). Without treatment, most subjects with complex CHD will die in the first year of life, with very few reaching adulthood (11, 16).

## Risk Factors of Congenital Heart Disease

**Figure 1:**
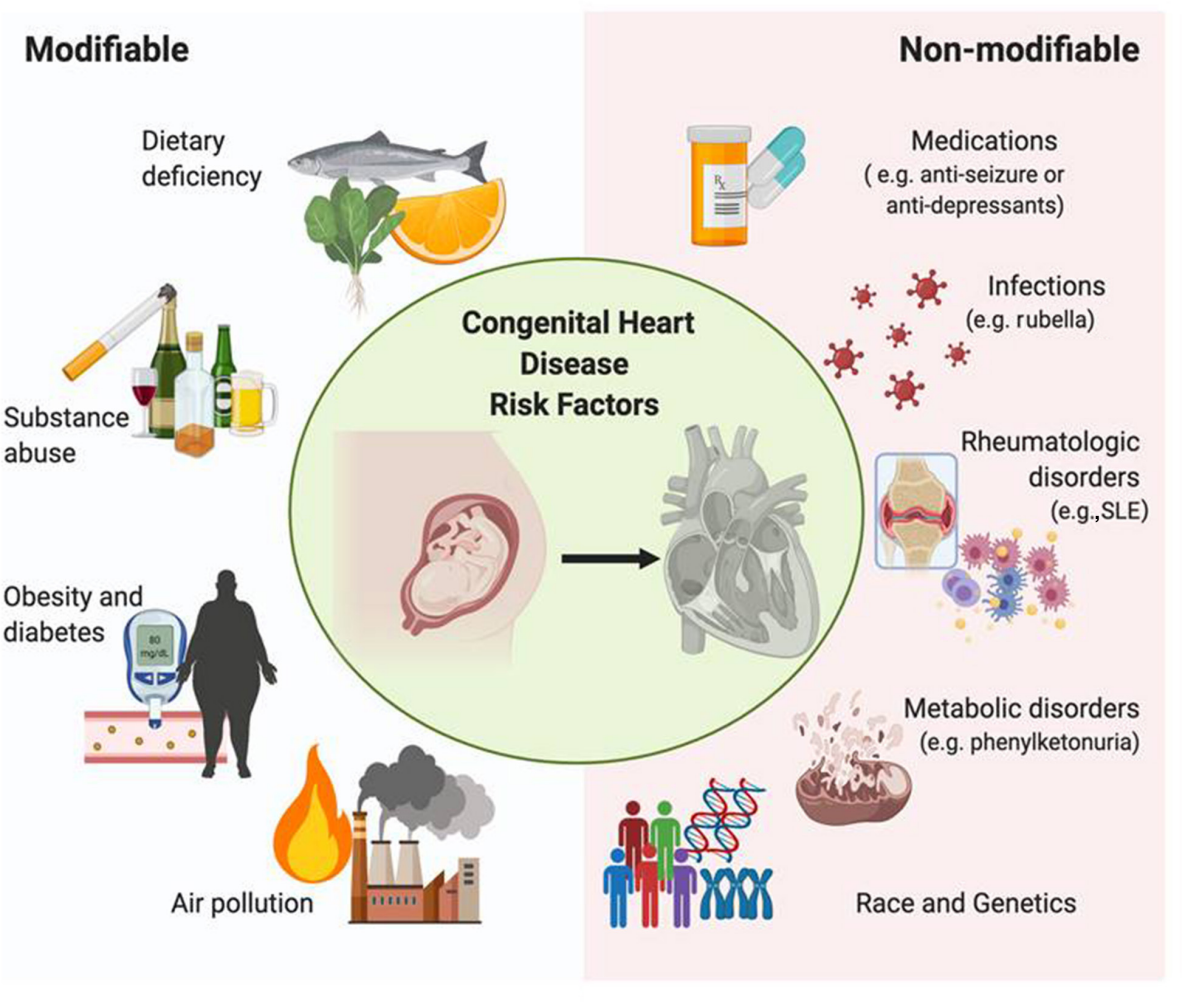
Risk factors of congenital heart disease. Modifiable risk factors associated with congenital heart disease include diet, obesity, substance abuse, and air pollution, whereas non-modifiable risk factors include rheumatologic disorders, genetic variants, specific medications, and infection. Each factor influences the development of this disease throughout pregnancy, effectively comprising the etiology of CHD and irreversibly altering the morphology of the fetal heart.

There are several risk factors contributing to CHD, including genetic/familial contributors as well as environmental/non-genetic factors promoting CHD development (17). Prenatal maternal conditions or exposures associated with an increased risk for CHD may be further categorized into modifiable vs. non-modifiable. Examples of modifiable risk factors include maternal dietary deficiency, substance abuse, obesity/diabetes, and air pollution. Examples of non-modifiable risk factors include maternal rheumatologic disorders, genetics, medications, metabolic disorders, and infections (e.g., rubella) (Figure 1).

### Modifiable Risk Factors

Studies have shown that daily folic acid deficiency during the pre- and peri-conception periods can be a CHD risk factor (17). Moderate maternal vitamin D deficiency has also been shown to significantly increase the risk of offspring developing CHD (18, 19). Additionally, more recent studies show that embryonic hypoxic exposure, caused by maternal smoking, increases the risk of CHD (18, 20, 21). Furthermore, maternal alcohol consumption, which can lead to the development of fetal alcohol syndrome disorder (FASD), is a commonly known risk factor for CHD. Heart defects such as VSD, ASD, and conotruncal defects occur quite frequently in FASD, with an estimated 67% of cases reported with CHD (18, 22, 23). Gestational diabetes is one of the most influential risk factors for CHD (24), increasing the risk by 3- to 5-fold (25). Poorly controlled maternal diabetes creates an unfavorable environment for embryonic development, exposing the fetus to elevated blood glucose levels, which is a major teratogen for diabetic embryopathy (25). Maternal diabetes induces cardiac malformation before the seventh week of gestation (26) with frequent cardiovascular malformations such as laterality and looping defects, including transposition of the great vessels, ASDs, VSDs, and HLHS (13, 26–28). Finally, multiple studies have suggested a link between air pollutants exposed during first trimester and the risk of VSD, ASD, and PDA. In 2013, Agay-Shay et al. evaluated the connection between maternal exposure to air pollution and congenital heart defects, finding an association between at least six separate cardiac anomalies with exposure to one of six major pollutants (29–36).

### Non-modifiable Risk Factors

Maternal exposure to pesticides and some therapeutic agents such as anti-seizure medications, thalidomide, and indomethacin tocolysis have been known to contribute to CHD development (26, 37). Additionally, potential confounding effects have been observed in studies evaluating the effect of maternal antidepressant use, specifically selective serotonin reuptake inhibitors (SSRIs) (17). Several studies also suggest that maternal viral infection in early pregnancy, such as rubella or cytomegalovirus, is associated with significantly increased risk of CHD. Maternal comorbidities such as rheumatologic disorders (e.g., systemic lupus erythematous) and metabolic disorders (e.g., phenylketonuria) have also been shown to increase the risk of CHD.

Moreover, accumulating data suggest racial and ethnic disparities in multiple key outcome measures for patients with CHD (38). Multiple studies have confirmed that when compared with their Caucasian counterparts, the incidence of all CHD in black infants was ~50% higher and also attributed to more severe and complex CHD types (39). Additionally, substantial data indicate that infant and child mortality from CHD is consistently higher among blacks when compared to Caucasians, which has been a reoccurring trend (40). In a 2003–2006 analysis of neonatal mortality due to CHD, the Centers for Disease Control and Prevention found that black infants had 20% higher mortality than Caucasian infants (38, 41). The precise reason for this disproportional rate of CHD mortality among blacks and Hispanics remains unknown, but may be correlated to underlying genetic variations specific to minority genomes, amongst other potential causes.

## Genetics of CHD

Multiple clinical, epidemiological, and embryological studies have acknowledged the significance of genetic factors in CHD etiology (12). Although a growing number of genetic contributors for CHD have been discovered, the etiology for the majority of CHDs remains unknown (25). Recent progress made possible by new molecular biology methods has identified some of the genes responsible for CHDs (12). Furthermore, previous studies positing a multifactorial inheritance hypothesis for the etiology of CHDs (i.e., *via* combined effects of multiple genes interacting with environmental and/or stochastic factors) may be further investigated using modern techniques (12, 42, 43).

The genetic etiologies of CHD are heterogeneous and include chromosomal as well as Mendelian factors (44). Previously CHD was primarily understood in the context of chromosomal abnormalities. Chromosomal anomalies are responsible for about 8–10% of presenting cases of CHD (17, 45). Chromosomal aneuploidy was the first identified genetic cause of syndromic CHD, accounting for a significant proportion of CHD (46). Chromosome anomalies also represent the most prevalent association, being diagnosed more frequently in patients with CHD than control subjects (12). Investigation into the genetic component for CHD was initiated based on their recurrence in families and by studies showing a correlation between CHD and inherited microdeletion syndromes (6). Previous research found that chromosomal aneuploidy is a genetic cause of syndromic CHD responsible for a large proportion of CHD (46). The most common chromosomal aneuploidy causing CHD is Down syndrome due to trisomy of chromosome 21 and partial trisomy 21 (i.e., translocation, mosaicism) (46). It is estimated that 50% of the patients with trisomy 21 (T21) and Turner syndrome (TS) have CHD (47). Similarly, other syndromic diseases such as Velo-cardio-facial syndrome/DiGeorge syndrome (VCFS/DGS), the most common micro-deletion disorder in humans, are characterized by craniofacial, parathyroid, and thymic defects as well as cardiac outflow tract malformations (48).

Over the past decade, remarkable advances in genetic sequencing technologies have enabled more rapid discovery of new genes contributing to CHD that allowed healthcare researchers to better understand the genetic basis of CHD (49, 50). For instance, next-generation sequencing (NGS) enables quick analysis of large amounts of genetic information (46, 51, 52). Bioinformatic analysis is vital to processing and analyzing the resultant biological data. Single-nucleotide polymorphisms (SNPs) represent changes in single nucleotides found in coding or non-coding regions of the genome (46). So far, several SNPs have been identified in the development and progression of CHD (46). For example, a study conducted on 114 CHD patients in 2001 by Junker et al. reported that MTHFR 677TT genotype was associated with CHDs such as pulmonary valve stenosis, coarctation of the aorta, HLHS, and aortic valve stenosis (46, 53). *De novo* mutations represent another genetic variant arising during embryogenesis that are known to influence CHD. In 2020, Homsy et al. used exome sequencing to identify an excess of protein-damaging *de novo* mutations in genes highly expressed in the developing heart (54).

Another useful advance is the identification of copy number variations (CNVs). CNVs of DNA sequences are sub-chromosomal changes resulting in a large deletion or amplification of DNA segments due to inappropriate recombination that leads to alterations in genes (46). CNVs are important because subtle variations in the number of copies of genes can significantly affect the development of cardiovascular disease (55). CNVs of various sizes can be identified by cytogenetic techniques such as comparative genomic hybridization (CGH) or multiplex ligation-dependent amplification (MLPA) (55), which can be used for detecting CNV in patients with isolated CHD (56).

### Genetic Link to Racial and Ethnic Disparity

The term “race,” in its traditional genetic conceptualization, is often determined through patterns of human variation reflected from our evolutionary history to serve as a definitive measure (57). Individuals belonging to different “races” are assumed to differ at the genome level (57). The assumption of “race” is an essential prerequisite when considering differences and commonalities among patients or groups of patients (57, 58). Genetic variations that determine phenotypic differences among individuals may also influence disease development, including cardiovascular disease states.

While studies have suggested that racial and ethnic differences may influence the prevalence and outcomes of CHD, the precise genetic and/or environmental causation is not well-established (1, 59, 60). It is possible that certain variants or CHD-susceptible genetic loci are more prevalent in certain ethnic or racial groups (59, 60). However, the temporal and racial variations in CHD occurrence remain poorly understood, highlighting the necessity to study further its genetic and environmental determinants (60). Additionally, racial and ethnic variations that influence CHD development may also be attributed to socioeconomic differences, cultural factors, lifestyle variation, and other factors that indicate environmental exposures (60). Resultant disparities in maternal health can impact the fetal environment and lead to birth defects, which may be partly explained by epigenetic mechanisms such as posttranslational modifications of histones, DNA methylation, and non-coding RNAs (25, 61, 62). In a recent study, Basu et al. reported an epigenetic mechanism underlying the gene-environment interaction between Notch1 haploinsufficiency and maternal diabetes mellitus that leads to CHD (25).

## Use of iPSCs to Model CHD

A compelling new paradigm in cardiovascular disease modeling is the use of induced pluripotent stem cells (iPSCs) and their differentiated cardiovascular cells to establish *in vitro* models of human physiology (63). First introduced in 2007, human iPSCs have revolutionized biomedical research by improving disease modeling and interrogation of drug response and toxicity, as well as generating a variety of cell types for therapeutic transplantation, amongst other advances (64, 65). Patient-specific iPSCs, especially in combination with advanced NGS technologies, are crucial in accelerating the investigation of molecular mechanisms for cardiovascular disorders while also helping to identify novel therapeutic targets for these diseases (64). A significant advantage is that iPSCs can be differentiated into various cell types such as cardiomyocytes (iPSC-CMs), endothelial cells (iPSC-ECs), and cardiac fibroblasts (iPSC-CFs), making it possible to study human genetics and proteins in their native cellular context (63).

Another benefit of using iPSCs derived from adult somatic cells is that they can be tailored to the unique individual genetics of patients. This generates novel insights into the molecular mechanisms of heart disease that may allow clinicians to deliver patient-specific pharmacological, genetic and cellular therapies in the future (66). In 2020, Miao et al. utilized scRNA-seq analysis with patient-specific iPSC-ECs and human fetal heart tissue to reveal endocardial functional defects and aberrant endocardium-myocardium crosstalk in HLHS (15). This innovative study focused on mutations in a transcription factor (ETS) and a chromatin remodeler (CHD7), identifying a large, downstream gene network that was differentially expressed in control vs. HLHS endocardial cells (15). Another study by Paige et al. observed a drastic impairment in contractility of HLHS iPSC-CMs in addition to associated changes in gene expression that significantly overlapped prior studies of human heart failure (67). Using iPSC-CMs they discovered 3 gene sets that were identified as molecular coordinators in heart failure (i.e., local, pathway, and central coordinators) (67). Finally, Kitani et al. provided genome-wide transcriptomic profiles of iPSC-CMs that were established from 5 patients with single ventricle disease (SVD) (including 1 HLHS, 2 tricuspid atresia, 1 double-outlet right ventricle, 1 double-inlet left ventricle) vs. five patients with non-syndromic ToF (5). They discovered that both SVD iPSC-CMs and ToF iPSC-CMs express unique transcriptomes compared with non-CHD iPSC-CMs. These and other studies provide growing evidence for the effectiveness of utilizing iPSCs to model disease states and develop novel therapeutic regimens (Table 1).

**Table 1:** iPSC-based studies to model CHD.

**References**	**CHD studied**	**Cell type**	**Experimental approach**	**General findings**
(5)	• SVD • ToF	• SVD iPSC-CMs • ToF iPSC-CMs	Provided genome-wide transcriptomic profiles of iPSC-CMs derived from patients with CHD.	SVD iPSC-CMs and ToF iPSC-CMs showed distinctive transcriptomes compared with non-CHD iPSC-CMs.
(15)	HLHS	HLHS iPSC-ECs	Utilized scRNA-seq analysis with iPSC-ECs to reveal endocardial defects associated with HLHS.	Decreased expression of FN1 is associated with endocardial and myocardial dysfunction in HLHS.
(67)	HLHS	HLHS iPSC-CMs	Performed functional assays with HLHS iPSC-CMs and utilized scRNA-seq analysis to identify genes associated with impairment in contractility in patients with HLHS.	Three gene sets were identified as molecular coordinators in heart failure in association with HLHS.
(68)	HLHS, hypoplasia of ascending aorta and left ventricle	HLHS iPSC-CMs	Performed NGS on a multigenerational family with CHD identifying a variant in MYH6 gene. Using their iPSC-CMs, investigated cardiogenesis influenced by MYH6 variants.	Clinical outcomes showed reduced survival rates among HLHS subjects with the damaging MYH6 variant. *In vitro* correlation showed impaired cardiogenesis.
(69)	Atrial and ventricular septal defects, and cardiomyopathy	SVD iPSC-CMs	Applied systems-level approach to investigate GATA4 roles in human cardiac development and function.	The GATA4-G296S mutation disrupts TBX5 recruitment, along with dysregulation of genes, leading to some of the phenotypic abnormalities associated with CHD.

*CHD, congenital heart disease; FN1, fibronectin 1; GATA binding protein 4; HLHS, hypoplastic left heart syndrome; iPSC-CMs, induced pluripotent stem cell-derived cardiomyocytes; iPSC-ECs, induced pluripotent stem cell-derived endothelial cells; MYH6, myosin heavy chain 6; NGS, next-generation sequencing; scRNA-seq, single cell RNA sequencing; SVD, single ventricle disease; ToF, tetralogy of Fallot; TBX, T-box transcription factor 5*.

### Modeling Racial Influence of CHD Using iPSCs

Patient-specific iPSCs help make it possible to analyze causal relationships in specific variants identified *via* SNP or CNV that are most prevalent among minorities (i.e., blacks and Hispanics). In 2016, Tomita-Mitchell et al. performed NGS on a multigenerational family with a high prevalence of CHD, identifying a rare variant in the α-myosin heavy chain (MYH6) gene (68). Additionally, Glessner et al. identified a loss-of-function mutation in ETS1 in patients with a hypoplastic left ventricle and other features found in Jacobsen syndrome (15, 70). Future advances should be directed toward identifying specific genetic variants that may be disproportionately affecting blacks and Hispanic cohorts, consequently influencing the severity of CHD.

Traditionally, minorities are not as well-represented in clinical trials or drug development compared to their Caucasian counterparts, a gap that may be addressed at least partially by using iPSCs to help identify drug or therapeutic applications effective in these populations. Various studies have shown the value of iPSCs in elucidating the molecular and cellular mechanisms of cardiac arrhythmias in disease states while providing a robust platform for the development novel drugs for clinical therapy (66). However, to date, little research has made the connection between racial disparities among patients with CHD that disproportionally affect minorities, and the full utility of iPSCs to address possible underlying genetic causal variants remains to be explored.

### Advancing Technologies

Using iPSCs to recapitulate a clinically relevant readout in a high throughput assay, researchers are now able to leverage chemical and functional genomics (e.g., siRNA and CRISPR screening) to better understand and interpret disease mechanisms for identifying novel therapeutic targets (63). The applications of these advanced technologies will expand rapidly in the future to improve our ability to understand and ultimately treat CHD (63). Elucidating the complex pattern of functional interactions between genomic variation and environmental exposure that regulate essential biological systems during heart development may help us better understand the correlation between CHD mortality and racial disparities. The ultimate goal is to create treatment platforms that will help reduce disproportionate mortalities among minorities (37).

Additionally, advances in genome-editing technologies have enabled biomedical researchers to precisely edit or introduce mutations in disease-causing genes to analyze the relative contribution of a single mutation on the severity of the disease phenotype (63). The utilization of genome-edited iPSC lines whose cardiovascular disease-associated mutations/variants are engineered into the same genetic background by endonuclease [i.e., zinc finger nucleases (ZFN), transcription activator-like effector nuclease (TALEN)] or palindromic repeat [i.e., clustered regularly interspaced short palindromic repeats (CRISPR)] is instrumental for generating libraries of disease-specific cardiomyocytes for drug testing and disease modeling (66, 71).

Another emerging technology that has the potential to enhance the management and understanding of CHD is machine learning. Future management and treatment of CHD will rely on increased understanding of the underlying mechanism of CHD, more precise early detection, and enhanced management strategies. Machine learning is an increasingly popular method to unearth underlying patterns in large datasets and in turn convert latent patterns into opportunities for early and precise predictions. Machine learning has already been utilized with CHD datasets to achieve early CHD diagnosis and to identify risk factors and optimal management strategies with great success. Using over 44,000 patient medical records for 10,019 patients, Diller et al. developed a deep learning model to predict primary clinical diagnosis, disease complexity, and optimal treatment regimen (69). In the future, CHD patients may be triaged at an early stage in an evidence-based manner and treatment strategies can be streamlined through *in silico* predictions. Further, machine learning is being used to identify and validate causes of CHD on a population level, in one instance elucidating the relationship between air pollutants and increased fetal CHD risk in pregnant women (69).

In addition to modeling the clinical progression of CHD, machine learning offers the potential, when combined with current CHD modeling platforms such as iPSCs and omics-technology, to unearth underlying genetic mechanisms of CHD. In particular, machine learning has been instrumental in analyzing many recently created cardiovascular single cell RNA sequencing (scRNA-seq) datasets (72). scRNA-seq are large datasets that benefit from the capability of machine learning models to learn from the datasets to identify inherent patterns and structure. Two classes of unsupervised machine learning models, dimensionality reduction and clustering techniques, have formed the basis of scRNA-seq analysis and visualization (73). The resolution of sc-RNAseq datasets combined with the data analysis power of machine learning have led to countless discoveries in CHD: scRNA-seq datasets have been used to create cell atlases of cardiac development (74), perform lineage reconstruction to delineate coronary artery development (75), and uncover mechanisms that regulate emergence and segregation of early cardiac lineages that form the heart (76). Specifically, when applied to iPSCs, machine learning has been used to identify key regulators in cardiac development from scRNA-seq of iPSC-CM differentiation (15, 69); and mechanisms of hypoplastic left heart syndrome from scRNA-seq of iPSC-endothelial cells (15).

## Conclusion

**Figure 2:**
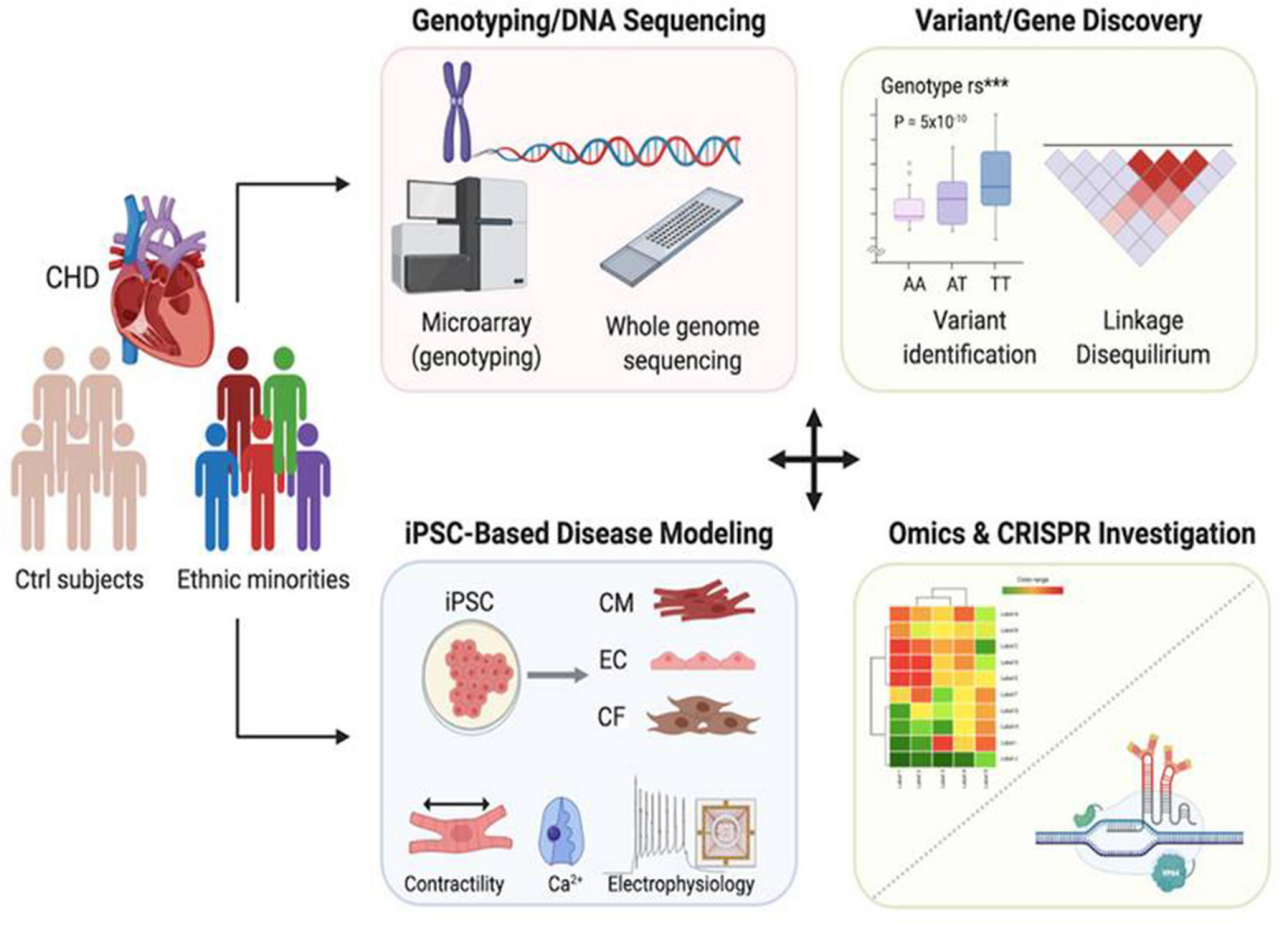
Applications of genomics and patient-specific iPSCs to reveal race-related genetic contribution to congenital heart disease. In combination with next generation sequencing and genome editing technology, iPSC-based disease modeling could be utilized to identify genetic variants that exist disproportionally within racial minority communities, thus providing a valuable tool for developing novel therapeutic treatment options to help those who have offspring suffering from CHD.

In summary, by utilizing advances in genomics and genetic technologies in combination with iPSCs, an innovative platform can be developed to address racial disparities in mortality rates among patients with CHD (Figure 2). By identifying novel genetic variants that are specific to minorities disproportionally afflicted with this disease, novel cardiovascular disease modeling systems can be designed to improve the manner in which CHD has been treated. By using the aforementioned gene editing technology as a therapeutic option, this could ultimately make it possible to identify the causes of racial disparities and find effective treatments to reduce them.

## Author Contributions

MM, J-WR, and JW contributed in drafting the manuscript. AZ, GL, and AR editing the manuscript. All authors contributed to the article and approved the submitted version.

## Conflict of Interest

The authors declare that the research was conducted in the absence of any commercial or financial relationships that could be construed as a potential conflict of interest.
